# Early Cell Fate Decisions of Human Embryonic Stem Cells and Mouse Epiblast Stem Cells Are Controlled by the Same Signalling Pathways

**DOI:** 10.1371/journal.pone.0006082

**Published:** 2009-06-30

**Authors:** Ludovic Vallier, Thomas Touboul, Zhenzhi Chng, Minodora Brimpari, Nicholas Hannan, Enrique Millan, Lucy E. Smithers, Matthew Trotter, Peter Rugg-Gunn, Anne Weber, Roger A. Pedersen

**Affiliations:** 1 Department of Surgery and Laboratory for Regenerative Medicine, University of Cambridge, Cambridge, United Kingdom; 2 Laboratoire de transfert de gènes dans le foie: applications thérapeutiques, Equipe Mixte Inserm U804, Université Paris XI Bâtiment Gregory Pincus, Le Kremlin Bic&être, France; 3 Wolfson Institute for Biomedical Research, University College London, London, United Kingdom; 4 Hospital for Sick Children, Toronto Medical Discovery Tower, Toronto, Ontario, Canada; Katholieke Universiteit Leuven, Belgium

## Abstract

Human embryonic stem cells have unique value for regenerative medicine, as they are capable of differentiating into a broad variety of cell types. Therefore, defining the signalling pathways that control early cell fate decisions of pluripotent stem cells represents a major task. Moreover, modelling the early steps of embryonic development in vitro may provide the best approach to produce cell types with native properties. Here, we analysed the function of key developmental growth factors such as Activin, FGF and BMP in the control of early cell fate decisions of human pluripotent stem cells. This analysis resulted in the development and validation of chemically defined culture conditions for achieving specification of human embryonic stem cells into neuroectoderm, mesendoderm and into extra-embryonic tissues. Importantly, these defined culture conditions are devoid of factors that could obscure analysis of developmental mechanisms or render the resulting tissues incompatible with future clinical applications. Importantly, the growth factor roles defined using these culture conditions similarly drove differentiation of mouse epiblast stem cells derived from post implantation embryos, thereby reinforcing the hypothesis that epiblast stem cells share a common embryonic identity with human pluripotent stem cells. Therefore the defined growth factor conditions described here represent an essential step toward the production of mature cell types from pluripotent stem cells in conditions fully compatible with clinical use ant also provide a general approach for modelling the early steps of mammalian embryonic development.

## Introduction

Human Embryonic Stem cells (hESCs) are pluripotent cells cultured from embryos at the blastocyst stage [1]. Their pluripotent status confers upon them the capacity to differentiate into a wide variety of cell types. However, generating fully functional cells from hESCs and achieving this goal using clinically-compatible conditions remain major challenges owing to the presence of undefined serum and cellular components in standard culture media and differentiation protocols. Indeed, widely-used methods are based on media formulations containing unknown factors and animal derived products in addition to serum such as Matrigel, and stroma cells [2], [3]. While modelling the early steps of embryonic development in vitro may provide the best approach for generating differentiated cells with native properties, devising protocols for differentiation of pluripotent stem cells is largely an empirical task. The earliest cell fate decisions during mammalian embryonic development are the specification of extra-embryonic tissues, starting with the trophectoderm and the primitive endoderm. Differentiation of definitive embryonic tissues occurs later, at the gastrulation stage, with the specification of the ectoderm, mesoderm and endoderm germ layers from which all the adult tissues are derived. Here, we analysed the effect of key developmental signalling pathways such as Activin/Nodal, FGF and BMP4 on early cell fate decisions of hESCs using an approach based on a fully chemically defined medium. This is resulted in the definition of the function of each pathway in the specification of extra-embryonic tissues and the primary germ layers. Based on these results, we developed and validated chemically defined culture conditions for achieving specification of hESCs into neuroectoderm and mesendoderm and into extra-embryonic tissues. Importantly, these culture conditions worked equally well to promote similar differentiation of epiblast stem cells (EpiSCs) derived from post-implantation mouse embryos [4], [5], although their effectiveness on mouse Embryonic Stem Cells (mESCs) was limited. Together, these results show that pluripotent mammalian stem cells can be induced to undergo early cell fate decisions in chemically defined medium supplemented with a minimal set of growth factors, thereby providing a general approach for modelling the transition between the pluripotent state and specification of the three germ layers during mammalian gastrulation. Finally, these defined culture conditions are devoid of animal products, thus they also represent a major step toward the generation of fully functional cell types compatible with future clinical applications.

## Results

### Approach used to identify growth factors driving differentiation of hESCs into ectoderm, mesoderm and endoderm in chemically defined medium

The function of Activin/Nodal, FGF, Wnt and BMP signalling pathways in the differentiation of the primary germ layers is highly conserved between amphibia [6], fish [7] and mammals [8]. Consequently, these growth factors appeared to be the best candidates to drive differentiation of hESCs. Accordingly, we undertook a systematic analysis of the effects of these four factors on hESC differentiation into neuroectoderm and mesendoderm in the absence of feeders cells, using a chemically defined medium (CDM) [9]. CDM is devoid of serum and is supplemented with insulin, transferrin and defined lipids to which was added either serum albumin or Polyvinyl Alcohol (PVA). The effect of inhibiting the Activin, FGF and PI-3-kinase signalling pathways was analysed in these conditions using respectively SB431542 [10], a pharmacological inhibitor of Activin/Nodal receptors, SU5402 [11], an inhibitor of FGF receptors, or LY294002 an inhibitor of PI-3-Kinase [12]. Briefly, H9 and hSF-6 hESC lines grown in CDM (containing bovine or human serum albumin) supplemented with Activin (10 ng/ml) and FGF (12 ng/ml) were plated on plastic dishes pre-coated with human fibronectin [13]. After 48 hours, Activin and FGF were replaced by other growth factors and inhibitors for varying periods. Expression of the pluripotency marker Oct-4, the pluripotency and neuroectoderm progenitor marker Sox2, the mesoderm marker Brachyury, and the endoderm marker Sox17 was analysed using immunostaining after 7 or 10 days of treatment (Tables 1 and 2). This approach enabled a rapid semiquantitative assessment of the combinational options involving the four growth factor pathways.

**Table 1:** Identification of culture conditions for inducing neuroectoderm and mesendoderm differentiation in CDM-BSA.

Conditions CDM	Pluripotency	Neuro	Meso	Endo
	0ct4	Sox2	Brachyury	Sox17
0	**L**	**H**	**VL**	**VL**
Activin 10 ng/ml+FGF 12 ng/ml (7 Days)	**H**	**H**	**0**	**0**
Activin 10 ng/ml+SU 10 µM (7 Days)	**H**	**H**	**L**	**L**
FGF 12 ng/ml+SB 10 µM (7 Days)	**0**	**H**	**0**	**0**
SB 10 µM+SU 10 µM	**0**	**0**	**0**	**0**
BMP4 50 ng/ml (7 days)	**0**	**0**	**0**	**0**
Acti 10 ng/ml+BMP4 50 ng/ml (7 days)	**0**	**0**	**0**	**0**
0 (4 or 5 days)+BMP4 50 ng/ml (3 days)	**0**	**0**	**0**	**0**
0 (4 or 5 days)+Activin 30 ng/ml+FGF 12 ng/ml (3 days)	**H**	**H**	**L**	**L**
0 (4 or 5 days)+Activin 30 ng/ml+BIO 5 µM (3 days)	**L**	**H**	**0**	**0**
SB 10 µM+BIO 5 µM (7 days)	**0**	**H**	**0**	**0**
0 (4 or 5 days)+Activin 30 ng/ml+SU 10 µM (3 days)	**L**	**L**	**L**	**VL**
BMP4 50 ng/ml+Activin 30 ng/ml (7 days)	**0**	**0**	**0**	**0**
SB 10 µM+BMP4 50 ng/ml+FGF2 12 ng/ml (7 days)	**0**	**L**	**0**	**0**
SB 10 µM (5 days)+BMP4 50 ng/ml+Activin 30 ng/ml (3 days)	**0**	**H**	**0**	**0**
SB 10 µM (5 days)+Acti 30 ng/ml (3 days)	**0**	**H**	**0**	**0**
SB 10 µM (5 days)+Acti 30 ng/ml+FGF 12 ng/ml (3 days)	**0**	**H**	**0**	**0**
SU 10 µM (3 days) Activin 30 ng/ml (4 days)	**H**	**H**	**L**	**L**
SU 10 µM (4 days) Activin 30 ng/ml (4 days)	**H**	**H**	**L**	**L**
SU 10 µM (5 days) Activin 30 ng/ml (4 days)	**L**	**L**	**L**	**L**

H9 hESCs were grown for different periods in CDM-BSA supplemented with different growth factors (Activin, FGF2 or BMP4) or chemical inhibitors [SB431542 (SB) for Activin/Nodal, SU5402 (SU) for FGF or BIO for GSK3β/Wnt]. Then, immunostaining analyses were used to determine the fraction of cells expressing the pluripotency marker Oct-4, the neuroectoderm marker Sox2, the mesoderm marker Brachyury, and the endoderm marker Sox17. The levels of marker-expressing cells were divided into five arbitrary categories: 0 for absence of expression, Very Low (VL) for <1% expressing cells, Low (L) for <5% expressing cells, Moderate (M) for >10% expressing cells, High (H) for >50% expressing cells. Indicated in blue are conditions allowing the generation of neuroectoderm (expressing Sox2) and in red those inducing mesendoderm (cells expressing Brachyury or Sox17).

**Table 2:** Identification of culture conditions for inducing neuroectoderm and mesendoderm differentiation in CDM-PVA.

Conditions CDM-PVA	Pluripotency	Neuro	Meso	Endo
	0ct4	Sox2	Brachyury	Sox17
Activin 10 ng/ml+FGF 12 ng/ml (7 Days)	**H**	**H**	**0**	**0**
Activin 10 ng/ml+SU 10 (7 Days)	**H**	**H**	**L**	**L**
FGF 12 ng/ml+SB 10 (7 Days)	**0**	**H**	**0**	**0**
SU 10 (3 days) Activin 30 ng/ml (4 days)	**H**	**H**	**L**	**L**
SU 10 (4 days) Activin 30 ng/ml (4 days)	**H**	**H**	**L**	**L**
SU 10 (5 days) Activin 30 ng/ml (4 days)	**H**	**H**	**VL**	**VL**
Activin 5 ng/ml+SU 10 (3 days) Activin 30 ng/ml (4 days)	**L**	**L**	**M**	**M**
Activin 5 ng/ml+SU 10 (3 days) Activin 100 ng/ml (4 days)	**L**	**L**	**L**	**H**
Activin 5 ng/ml+SU 10 (3 days) Activin 30 ng/ml+FGF 20 ng/ml+BMP4 10 ng/ml (4 days)	**VL**	**VL**	**H**	**H**

H9 hESCs were grown for different periods in CDM-PVA supplemented with different growth factors (Activin, FGF2 or BMP4) or chemical inhibitors SU5402 (SU) for FGF. Then, immunostaining analyses were used to determine the fraction of cells expressing the pluripotency marker Oct-4, the neuroectoderm marker Sox2, the mesoderm marker Brachyury, and the endoderm marker Sox17. The levels of marker-expressing cells were divided into five arbitrary categories: 0 for absence of expression, Very Low (VL) for <1% expressing cells, Low (L) for <5% expressing cells, Moderate (M) for >10% expressing cells, High (H) for >50% expressing cells. Indicated in blue are conditions allowing the generation of neuroectoderm (expressing Sox2) and in red those inducing mesendoderm (cells expressing Brachyury or Sox17).

### BMP4 drives differentiation of hESCs into a mixture of primitive endoderm and trophectoderm cells in chemically defined conditions

**Figure 1:**
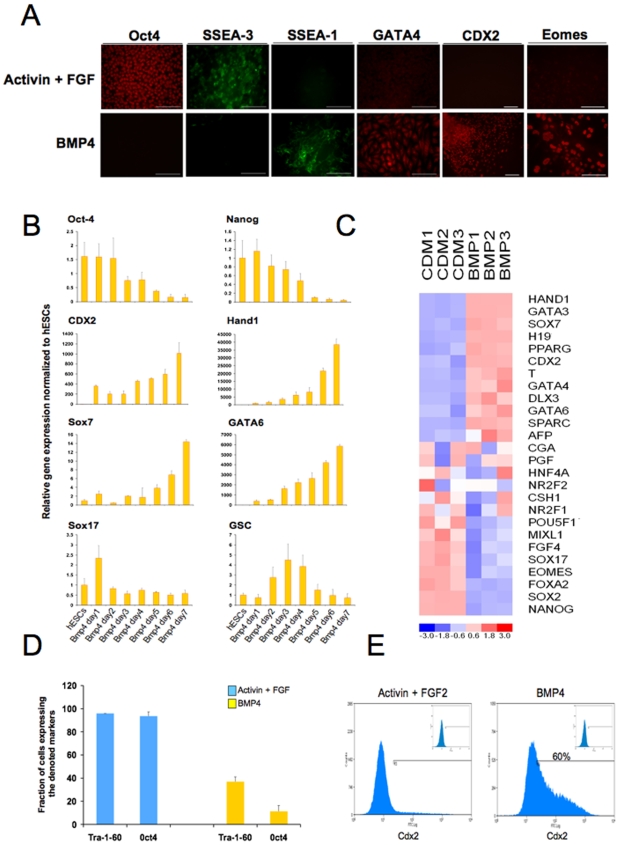
Generation of extra-embryonic tissues using BMP4. (A) Immunostaining analyses for the expression of pluripotency markers (Oct-4, SSEA-3), primitive endoderm markers (SSEA-1, GATA4), and trophectoderm markers (CDX2, Eomes) by H9 cells grown for 7 days in CDM+BMP4 10 ng/ml. Scale Bar 50 µM. (B) Dynamic expression of pluripotency markers (Oct-4, Nanog), trophectoderm markers (CDX2, Hand1), primitive endoderm markers (Sox7, GATA6) and definitive endoderm markers (Sox17, GSC) during differentiation of hESCs. H9 cells were differentiated following the protocol described above. Following the first day after plating, RNAs were extracted every day and expression of the denoted genes was analysed using Q-PCR. Normalized expression is shown as the mean±SD from two informative experiments. (C) A microarray gene expression heat map to compare human embryonic stem cells (ESC) grown in CDM and extra-embryonic cells generated in CDM (CDM) supplemented with BMP4 (BMP). For each gene (row) the heat map colours sample gene expression in units of standard deviation from the mean across all samples (columns). Up-regulation is coloured in shades of red and down-regulation in shades of blue according to the scale shown at the bottom of the heat map. (D) Fraction of cells expressing the pluripotency markers Tra-1-60 and Oct-4 after growth in the presence of BMP4 for 7 days. H9 cells were grown in CDM+BMP4 10 ng/ml, and then the fraction of cells expressing Oct-4, and Tra-1-60 was determined using FACS. H9 cells grown in CDM+Activin 10 ng/ml+FGF2 12 ng/ml were used as positive control. (E) Fraction of cells expressing CDX2 after extra-embryonic differentiation. H9 cells were grown in CDM+BMP4 10 ng/ml for 7 days, and then the fraction of cells expressing CDX2 was determined using FACS. H9 cells grown in CDM+Activin 10 ng/ml+FGF2 12 ng/ml were used as negative control.

Addition of BMP4 in CDM induced a rapid decrease in the level of expression of Oct-4 and SSEA-3 while increasing the expression of the differentiation marker SSEA-1 (Figure 1A) confirming that in contrast to mESCs, hESCs are not maintained as pluripotent cells by BMP4 signalling [14], [15]. Further studies including immuno-staining (Figure 1A), Real time PCR (Figure 1B), and microarray (Figure 1C) analyses revealed that BMP4 treatment also induced the concomitant expression of primitive endoderm markers including GATA4, GATA6, Sox7, CoupTFII and trophectoderm markers H19, CDX2, Eomesodermin, Hand-1 and hαCG (Figure 1A, 1B, 1C, 1D and data not shown). FACS analysis revealed that 90% of the cells grown for 7 days under these culture conditions had lost expression of the pluripotency markers Tra-1-60 and Oct-4 (Figure 1D), confirming the efficacy of BMP4 in inducing differentiation of hESCs. In addition, cells generated under these conditions did not express the mesendoderm markers Sox17 and GSC or the neuroectoderm marker Sox1 (data not shown). Importantly, immunostaining analyses showed that cells expressing the trophectoderm marker CDX2 did not co-express the primitive endoderm marker Sox7 (data not shown), indicating that these two cell types represent distinguishable outcomes. These results demonstrate that activation of BMP signalling in CDM is sufficient to drive differentiation of hESCs toward the two earliest extra-embryonic lineages that form in mammalian development. These results differ from the study by Xu and Thomson, which suggested that BMP4 drives differentiation of hESCs into a homogenous population of trophectoderm cells [15]. However, their experiments were performed on Matrigel and in medium containing serum and thus included undefined factors that could have interfered with the primitive endoderm-inducing effect of BMP4. This difference illustrates the importance of conducting cell fate studies in chemically defined conditions, where the identities of differentiation-inducing or inhibiting factors are known.

### Inhibition of Activin/Nodal signalling in the presence of FGF drives differentiation of hESCs into neuroectoderm progenitors

**Figure 2:**
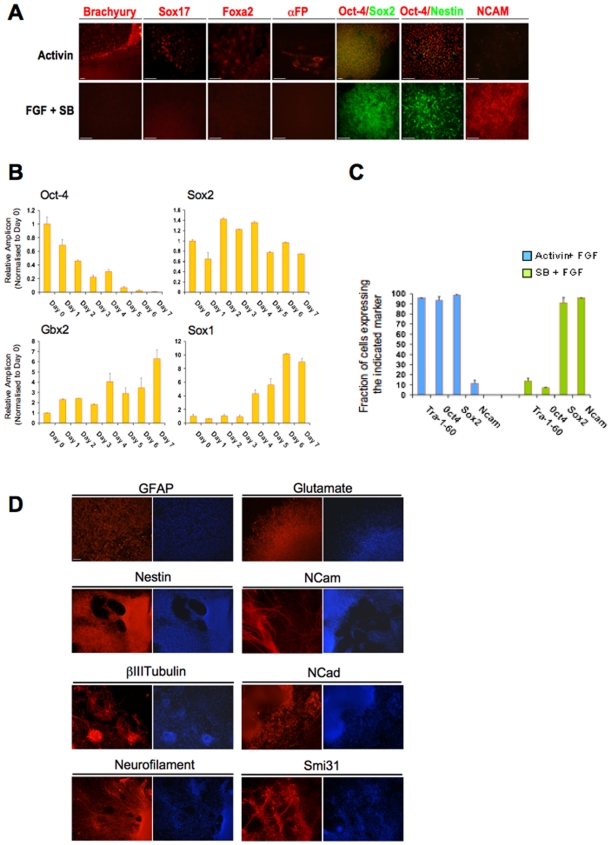
Generation of neuroectoderm precursors by inhibiting Activin signalling in the presence of FGF2. (A) Immunostaining analyses for the expression of a pluripotency marker (Oct-4), neuroectoderm markers (Sox2, Nestin, N-CAM) or mesendoderm markers (Brachyury, Sox17, FoxA2, αFP) by H9 cells grown for 7 days in CDM+Activin 100 ng/ml or in CDM+SB431542 10 µM+FGF2 12 ng/ml. Scale Bar 50 µM. Note homogenous expression of both Oct-4 and Sox2 in Activin (Yellow staining), but only Sox2 in FGF+SB (Green Staining). (B) Dynamic expression of a pluripotency marker (Oct-4) and neuroectoderm markers (Sox2, Sox1, Gbx2) during differentiation of hESCs. H9 cells were differentiated following the protocol described above. Following the first day after plating, RNAs were extracted every 2 days and expression of the denoted genes was analysed using Q-PCR. Normalized expression is shown as the mean±SD from two informative experiments. (C) Fraction of cells expressing the pluripotency markers Oct-4, Tra-1-60 and Sox2 and the neuronal markers Sox2 and N-CAM after growth in the presence of SB431542 for 7 days. H9 cells were grown in CDM+SB431542 10 µM+FGF2 12 ng/ml (SB+FGF), and then the fraction of cells expressing Oct-4, Sox2 and N-CAM was determined using FACS. H9 cells grown in CDM+Activin 10 ng/ml+FGF2 12 ng/ml (Activin+FGF) were used as control. (D) Expression of neuronal markers by fully differentiated neuroectoderm progenitors. H9 cells were induced to differentiate into neuroectoderm in CDM+SB431542 10 µM+FGF2 12 ng/ml. The resulting neuroectoderm progenitors were then grown as embryoid bodies (EBs) in non-adherent conditions for 14 days to allow further differentiation. The resulting EBs were then plated back on plastic and grown for 14 additional days before assessment. Nuclei are shown by Hoechst staining. Scale Bar 100 µM.

Our assessment of the four signalling pathways also showed that inhibition of Activin/Nodal signalling by the Alk4/5/7 inhibitor SB431542 (10 µM) systematically resulted in the loss of pluripotency markers including Oct-4, while expression of Sox2 was maintained (Figure 2A, 2B and 2C), confirming that inhibition of Activin signalling induces differentiation of hESCs toward neuroectoderm [16]. The addition of FGF2 (12 ng/ml) or BIO (5 µM), a GSK3β inhibitor that mimics Wnt activity [17], was not sufficient to maintain expression of Oct4 or to induce expression of the mesendoderm and endoderm markers Brachyury and Sox17 in the absence of Activin/Nodal activity (data not shown). Thus in the absence of Activin signalling, FGF and Wnt signalling could neither maintain the pluripotent status of hESCs nor drive their differentiation toward mesendoderm in these chemically defined culture conditions. Additionally, cells expressing the endoderm markers Sox17, FoxA2 and Alpha-Fetoprotein (αFP) were never observed following treatment with SB431542 (Table 1 and Figure 2A), confirming the importance of Activin/Nodal signalling in the commitment of pluripotent stem cells toward definitive endoderm [2], [18]. Finally, a combination of SB431542 (10 µM) and FGF2 (12 ng/ml) in CDM generated highly proliferative cells that expressed the neuroectoderm markers Sox1, Sox2, Gbx2, Nestin and NCam, but not the pluripotency markers Oct4, SSEA-3, Tra-1-60 or Nanog (Figure 2A, 2B, 2C, and data not shown). FACS analysis revealed that 95% of the cells grown for 7 days under these latter culture conditions maintained the expression of Sox2 (also an early neuroectoderm marker) and became positive for the pan-neuronal marker NCAM, as compared to only 10% displaying NCam in CDM supplemented with Activin and FGF (Figure 2C). Importantly, FGF signalling was necessary for neuroectoderm differentiation since addition of SU5402 in CDM supplemented with SB431542 essentially blocked this process (data not shown). Finally, when the Oct4(−)/Sox2(+) cells generated by a combination of SB431542 (10 µM) and FGF (12 ng/ml) treatment were grown as embryoid bodies (EBs) in non-adherent conditions in CDM for 15 days and then the EBs were returned to adherent culture conditions for 10 additional days, they produced progeny showing widespread expression of neuronal differentiation markers, including Nestin, NCAM, GFAP, Glutamate, N-cadherin, βIIITubulin, Neurofilament and Smi3 (Figure 2D). Together these results demonstrate that inhibition of Activin/Nodal activity in the presence of sustained FGF activity is sufficient to generate a population of neuroectoderm progenitors.

### A combination of Activin, BMP4 and FGF2 in a three step protocol drives differentiation of hESCs into mesendoderm

**Figure 3:**
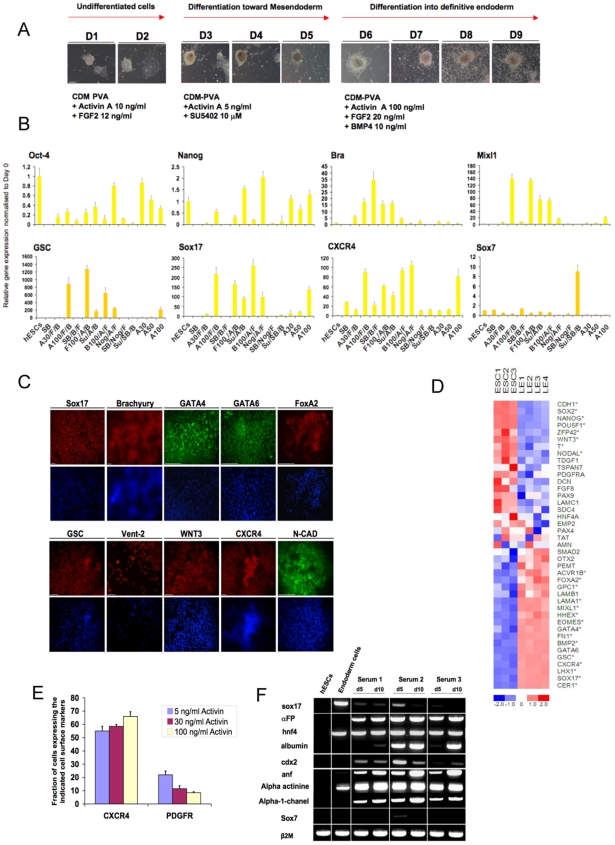
Generation of mesendoderm using a combination of Activin, FGF2 and BMP4. (A) Colony morphologies formed in response to the three-step protocol to differentiate hESCs into mesendoderm progeny. H9 cells were grown for 2 days in CDM/PVA+Activin 10 ng/ml+FGF2 12 ng/ml, then for 72 hours in CDM/PVA+SU5402 10 µM+Activin 5 ng/ml. The next 4 days, cells were grown in CDM/PVA+Activin 30 ng/ml+FGF2 20 ng/ml+BMP4 10 ng/ml. Images of the same colonies were captured every day for 9 days. Scale Bar 200 µM. (B) Effect of different combinations and doses of Activin, FGF2 and BMP4 on the differentiation of hESCs grown in CDM/PVA. Following the third day in CDM/PVA+SU5402 10 µM, H9 cells were induced to differentiated in CDM/PVA supplemented with different combination of growth factors. RNAs were extracted after 3 days and expression of the denoted genes was analysed using Q-PCR. Normalized expression is shown as the mean±SD from two informative experiments. hESCs grown in CDM+Activin+FGF or differentiated in CDM+SB431542+FGF2 were used as negative controls. (C) Expression of specific markers for mesendoderm in hESCs differentiated in CDM/PVA supplemented with Activin, FGF2 and BMP4 in the three step protocol. Nuclei are shown by Hoechst staining. Scale Bar 100 µM. (D) Microarray gene expression heat map to compare human embryonic stem cells (ESC) grown in CDM supplemented with Activin and FGF and mesendoderm cells generated in CDM/PVA supplemented with Activin, FGF and BMP4 (LE). Up-regulation is coloured in shades of red and down-regulation in shades of blue according to the log z scale shown at the bottom of the heat map. Gene names marked with an asterisk denote genes that pass a significant differential regulation threshold. (See Material and Methods). (E) Fraction of cells expressing the definitive endoderm marker CXCR4 and the mesendoderm/mesoderm marker PDGFαR after induction of differentiation in CDM PVA in the presence of increasing doses of Activin. H9 cells were differentiated following the three step protocol described above in the presence of Activin (30 or 100 ng/ml). Fraction of cells expressing CXCR4 or PDGFαR was determined using FACS 8 days after plating. (F) RT-PCR analyses for the expression of liver markers (Albumin, HNF4, αFP), gut marker (CDX2) and cardiac markers (ANF, α Actinin, α-1 Channel) in endoderm progenitors grown in media containing serum. Endoderm progenitors generated using the three step protocol were differentiated in media containing three different FBS lots. RNAs were extracted after 5 and 10 days of differentiation and the expression of genes expressed was analysed using RT-PCR.

Another important outcome of the approach was to demonstrate the effect of Activin on mesendoderm differentiation in chemically defined conditions. Indeed, Activin has been shown in several studies to be able to generate mesendoderm cells from mouse and human ESCs [2], [3]. We found that high doses of Activin (30 or 100 ng/ml) in CDM maintained a predominant population of cells expressing the pluripotency markers Oct-4 and Nanog while also inducing the expression of the definitive endoderm markers Sox17 and CXCR4 in some cells (Figure 3B and data not shown), suggesting that Activin signalling is permissive but not sufficient for differentiation of hESCs toward the mesendoderm lineage. The insufficiency of Activin for mesendoderm differentiation in CDM differs from its effect in previously published approaches that used serum, stroma cells and complex extra-cellular matrices, all of which contain unknown factors capable of influencing growth factor induced differentiation. In agreement with this interpretation, we found that a combination of Activin, FGF2 and BMP4 signalling efficiently induced mesendoderm differentiation using a three step protocol (Figure 3A). In the first step, hESCs grown initially in CDM+Activin+FGF2 were plated for 48 hours on fibronectin in CDM-PVA medium containing Activin (10 ng/ml) and FGF2 (12 ng/ml). This first step allowed the hESC colonies to attach properly, and as expected cells continued to express high levels of Oct4 but not Brachyury or Sox17 (data not shown). In the second step, SU5402 (10 µM) and a lower dose of Activin (5 ng/ml) were added for three days. Addition of SU5402 reduced adhesion of the hESC colonies, which began to form compact aggregates of cells (Figure 3A) expressing Oct4 and low levels of Brachyury but not Sox17 (data not shown). These observations suggest that SU5402 acts as a pre-differentiation step in which neuroectoderm differentiation was prevented by the inhibition of FGF signalling and by the presence of Activin. In the third step, a combination of BMP4 (10 ng/ml), FGF2 (20 ng/ml) and Activin (30 or 100 ng/ml) was added, leading to spreading and differentiation of the colonies (Figure 3A). After 24 hours in these conditions, a majority of cells expressed high levels of Brachyury, while Oct4 expression declined further (data not shown). During the following three days, the number of Sox17 expressing cells increased progressively, while Brachyury or Oct4 expressing cells almost disappeared (data not shown and Figure 3B). Q-PCR (Figure 3B) and immunostaining (Figure 3C) analyses showed that these cells expressed markers of definitive endoderm including CXCR4, Goosecoid, Mixl1, FoxA2, GATA4, GATA6, Vent-2, WNT3, and N-Cadherin. Moreover, transcripts of Sox7, a gene specifically expressed in extra-embryonic endoderm, were not detected in this population of cells by Q-PCR (Figure 3B). These observations were confirmed by microarray analyses showing that the gene expression profile characteristic of hESCs (high Oct-4, Nanog, Sox2, Cripto and Nodal, etc.) was replaced with a profile characteristic of definitive endoderm (high Sox17, CXCR4, FoxA2, GATA4 and GATA6, etc.) (Figure 3D) when hESCs were subjected to this protocol. Taken together these findings show that modulation of Activin, FGF and BMP signalling can lead to differentiation of mesendoderm in chemically defined conditions.

We further explored the mesodermal and endodermal composition of the cells induced to differentiate using this three step protocol. FACS analysis revealed that the proportion of cells expressing the definitive endoderm surface marker CXCR4 [2], [18] or PDGFαR, a surface marker expressed in mesendoderm and mesoderm during in vitro differentiation of mouse ESCs [19], depended on the dose of Activin added at the third step of the protocol (Figure 3E). In the presence of a high dose of Activin (100 ng/ml), 70% of the cells expressed CXCR4 while only 7% expressed PDGFαR. On the other hand, in the absence of exogenous Activin, only 55% of the cells became positive for CXCR4 while 21% of the cells expressed PDGFαR. This observation was confirmed by Q-PCR analyses showing that the highest expression of definitive endoderm markers (Sox17, Mixl1, GSC) was obtained with the highest dose of Activin (100 ng/ml). On the other hand, inhibition of Activin signalling by SB431542 during the third step of the protocol abolished expression of definitive endoderm markers and resulted in the highest level of expression for the mesendoderm/mesoderm marker Brachyury (Figure 3B). Higher doses of BMP4 (100 ng/ml) or FGF (100 ng/ml) did not increase the expression of mesendoderm markers, while inhibition of BMP signalling by Noggin in the presence of Activin and FGF2 resulted in persistent expression of pluripotency markers (Oct-4, Nanog) (Figure 3B). In addition, inhibition of FGF signalling by SU5402 reduced cell proliferation without decreasing expression of mesendoderm markers (Figure 3B and data not shown). Taken together, these results demonstrate that BMP4 signalling acts as an inducer of mesendoderm differentiation whereas the intensity of Activin signalling influenced the cell fate choice between mesoderm and endoderm [18], [20]. Finally, we tested the capacity for further differentiation of these mesendoderm progenitors by using a medium containing Foetal Bovine Serum (FBS). In these conditions endoderm and mesoderm cells further differentiated into diverse cell types expressing liver (Albumin, αFeto Protein, HNF4), gut (CDX2) and cardiac markers (Atrial natriuretic factor (ANF), α Actin, α1 Channel) markers (Figure 3F). Additionally, beating cells were often observed in these culture conditions (data not shown). Together these results confirm that the mesendoderm progenitors generated with the three step protocol were capable of differentiating further into more mature endodermal and mesodermal derivatives. Finally, equivalent results using the three step protocol were obtained with both of the hESC lines used in this study (H9 and hSF-6) (data not shown), thereby demonstrating a robust approach for driving differentiation of hESCs toward the mesendoderm lineage in chemically defined conditions.

### The signalling pathways controlling differentiation of hESCs are shared by mouse EpiSCs but not by mESCs

**Figure 4:**
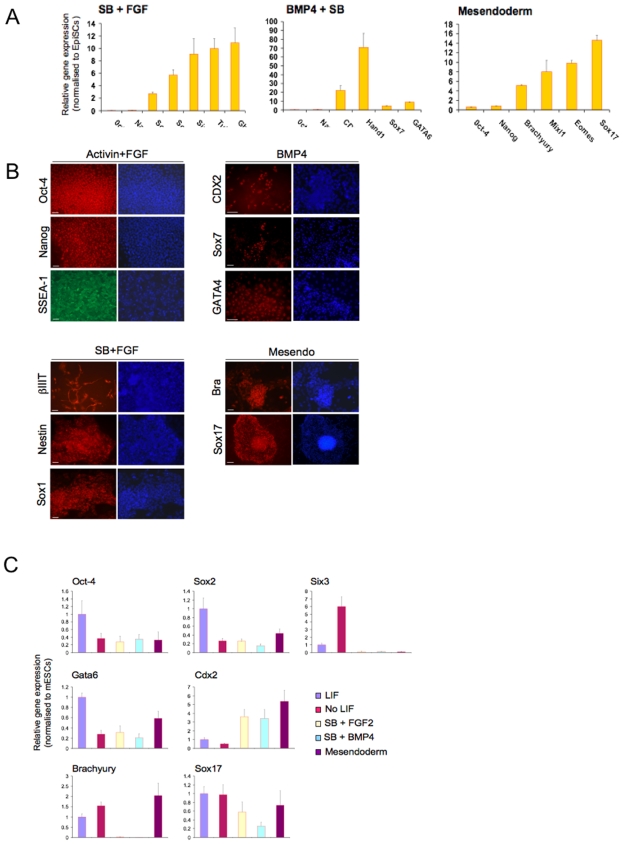
Contrary to mESCs, EpiSCs are responsive to culture conditions controlling differentiation of hESCs into extra-embryonic tissues, neuroectoderm and mesendoderm. (A) Expression of pluripotency markers (Oct-4, Nanog) and neuroectoderm (Sox2, Sox1, Six3, Tuj1 and Gbx2), extra-embryonic markers (Cdx2, Hand1, Sox7, GATA6) and mesendoderm markers (Brachyury, Mixl1, Eomes, Sox17) during differentiation of mouse EpiSCs using the culture conditions developed with hESCs. EpiSCs were differentiated following the conditions described above for hESCs (SB+FGF for neuroectoderm, BMP4 for extra-embryonic tissues, three step protocol for mesendoderm). Following the ninth day after plating, RNAs were extracted and expression of the denoted genes was analysed using Q-PCR. Normalized expression is shown as the mean±SD from two informative experiments. (B) Expression of pluripotency markers (Oct-4, Nanog, SSEA-1), extra-embryonic markers (CDX2, Sox7, GATA4), neuroectoderm markers (Sox1, Nestin, βIII tubulin) and mesendoderm markers (Brachyury and Sox17) in EpiSCs differentiated using the conditions developed for hESCs. Expression of the genes denoted was analysed by immuno-fluoresence analyses. Nuclei are shown by Hoechst staining. Scale Bar 100 µM. (C) Expression of pluripotency markers (Oct-4), extra-embryonic markers (CDX2, GATA6), neuroectoderm markers (Sox2, Six3) and mesendoderm markers (Brachyury and Sox17) in mESCs differentiated using the method developed with hESCs. mESCs were differentiated in CDM as described for hESCs and then the expression of the genes denoted was analysed by Real-Time PCR. Normalized expression is shown as the mean±SD from three experiments.

We and others recently derived pluripotent stem cells (EpiSCs) from the epiblast of post-implantation mouse embryo [4], [5]. These novel pluripotent embryonic stem cells share many properties with hESCs including their low efficiency of clonal propagation, their dependence on Activin signalling to maintain their pluripotency, and their capacity to differentiate into extra-embryonic tissues including trophectoderm in response to BMP4 [4]. Consequently, we analysed the efficiency of the chemically defined culture conditions described above to induce differentiation of mEpiSCs into neuroectoderm, extra-embryonic tissues, and mesendoderm. Inhibition of Activin/Nodal signalling by SB431542 (10 µM) systematically resulted in acute, widespread loss of pluripotency markers including Oct-4 and Nanog, while increasing expression of Sox2, Sox1, Gbx2, Six3, and Tuj1 (Figure 4A and 4B). This suggests that similarly to hESCs, inhibition of Activin signalling induces differentiation of mEpiSCs into neuroectoderm. As compared to hESCs, induction of extra-embryonic differentiation in EpiSCs by addition of BMP4 (10 ng/ml) in CDM in the absence of Activin and FGF was less efficient. Indeed, loss of pluripotent cell markers was observed only after 2 weeks of culture in CDM+BMP4 as compared to 5 days for hESCs. However, the effect of BMP4 on differentiation was strongly increased by adding the Activin/Nodal receptor inhibitor SB431542. A combination of BMP4 and SB431542 resulted in a rapid decrease in the expression of Oct-4 and Nanog, while increasing the expression of trophectoderm markers (Cdx2, Hand1, αCG, H19, Eomes) and the expression of primitive endoderm markers (Gata4, Gata6, Sox7) (Figure 4A and 4B) [4]. This confirms that similarly to hESCs, BMP4 signalling drives differentiation of EpiSCs into extra-embryonic tissues. However, these observations also suggest that contrary to hESCs, the endogenous Activin/Nodal-like signalling activity in EpiSCs is sufficient to block differentiation of EpiSCs induced by exogenous BMP4. Finally, mEpiSCs were subjected to the three step protocol for mesendoderm induction developed for hESCs. This led to a pronounced decrease in pluripotency markers (Oct-4 and Nanog) and an increase in mesendoderm markers (Brachyury, Eomes, Mixl1, Sox17) (Figure 4A and 4B). Taken together, these results show that mEpiSCs can be induced to differentiate into neuroectoderm, extra-embryonic tissues, and mesendoderm using similar chemically defined culture conditions as hESCs.

We then analysed the efficiency of these culture conditions for driving differentiation of mESCs. All three culture conditions tested (SB431542+FGF2; BMP4; three step protocol) induced differentiation of mESCs, as shown by an abrupt decrease in their Oct-4 expression (Figure 4C). However, these experiments were performed in the absence of LIF and this condition alone was sufficient to induce differentiation of mESCs grown in CDM (Figure 4c). Furthermore, inhibition of Activin/Nodal signalling by SB431542 did not increase mESC expression of the neuroectoderm markers Sox2 and Six3, and addition of BMP4 did not induce a specific and significant increase in the expression of the extraembryonic tissue markers GATA6 and CDX2 (Figure 4C). Finally, when the three step protocol was applied to mESCs, there was only a marginal increase in Brachyury and no concomitant increase in Sox17 (Figure 4C), suggesting that mESCs were not able to progress toward the definitive endoderm lineage under these culture conditions. Importantly similar results were generated with two different mESC line (CGR8 kind gift of Prof. Austin Smith, and R1 kind gift of Prof. Andreas Nagy) confirming these observation were independent of the cell line used. Taken together, these observations show that the chemically defined growth factor conditions capable of driving differentiation of hESCs and mEpiSCs were not similarly effective with mESCs. This implies that the distinct embryonic origin or identity of different types of pluripotent stem cells define their responsiveness to differentiation induction, just as it does for maintenance of pluripotency. These results also support the hypothesis that hESCs more closely resemble pluripotent cells of post-implantation mouse embryos than pluripotent cells of the inner cell mass, since hESCs and EpiSCs react similarly to all the culture conditions tested here [4], [5].

## Discussion

### hESCs and EpiSCs provide convergent in vitro systems enabling the analysis of mechanisms controlling cell fate decisions during early mammalian development

**Figure 5:**
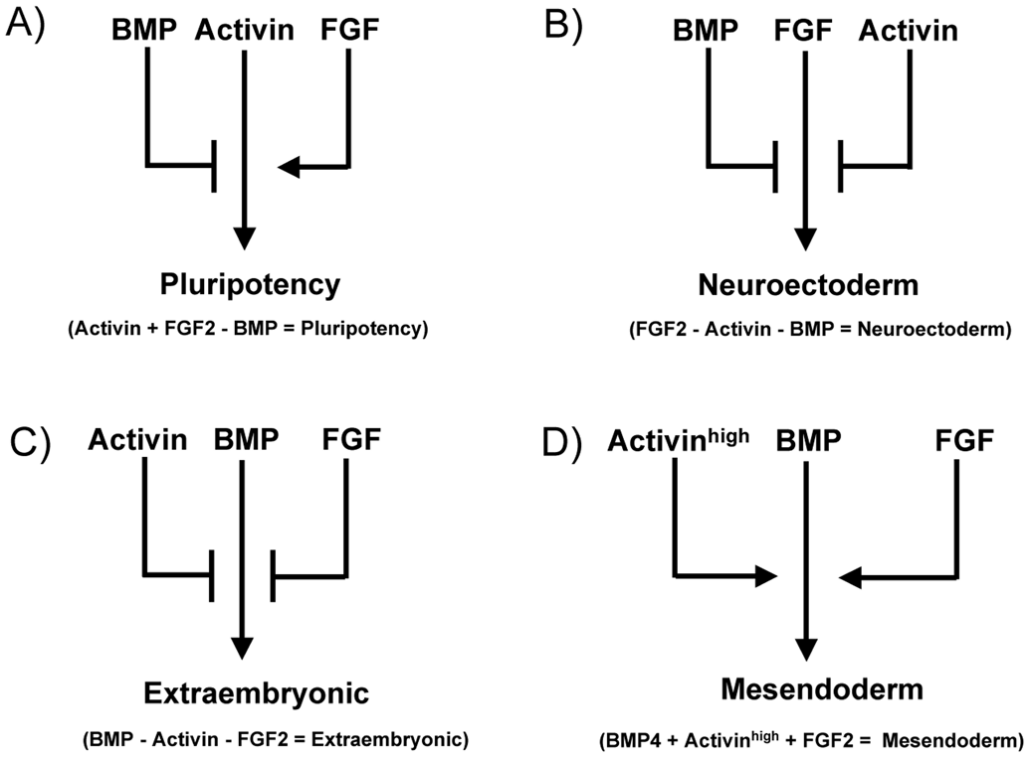
Cell signalling pathways controlling cell fate specification of pluripotent cells in vitro. A) Pluripotency of hESCs and mEpiSCs relies on Activin signalling and to a lesser extent on FGF signalling to maintain their pluripotent status. BMP signalling inhibition may be required to avoid extra-embryonic differentiation, depending on the level of endogenous BMP signalling activity of each cell type. B) Inhibition of Activin/Nodal signalling induces differentiation toward neuroectoderm in the presence of FGF2. C) BMP4 induces differentiation toward extra-embryonic tissues which is blocked by Activin and FGF2. D) BMP alternatively induces mesendoderm in cooperation with Activin (high dose) and FGF2. This model summarises results from hESCs and mEpiSCs and distinguish them from mESCs which remain pluripotent in LIF+BMP.

Taken together, our observations show that progenitors both for embryonic and extra-embryonic tissues (primitive endoderm and trophectoderm) resulting from the earliest cell fate decisions in mammalian development can be generated in chemically defined conditions from hESCs and EpiSCs. Moreover this can be achieved by modulating the activities of a small number of specific growth factors either through their addition to the medium or their pharmacological inhibition (for summary see Figure 5). Our systematic analysis clearly showed that inhibition of Activin/Nodal signalling in the presence of FGF2 was sufficient to initiate neuroectoderm specification by all the four types of pluripotent stem cells in vitro. While the importance of FGF signalling in neuroectoderm specification has been clearly established in chick and amphibian embryos and in mESCs [21]–[24], the function of Activin/Nodal in this cell fate choice in vertebrate embryogenesis has only recently been described. Overexpression of Nodal in hESCs blocks neuroectoderm differentiation of hESCs [25] whereas overexpression of the Nodal inhibitor Lefty promotes differentiation of hESCs toward neuroectoderm [16]. Interestingly, genetic studies have shown that inhibition of Nodal is also sufficient to induce neuroectoderm specification at a pre-gastrulation stage in the mouse embryo [21]–[24], [26]. Similarly, inhibition of Activin/Nodal receptor-mediated signalling is necessary for neural differentiation in Xenopus embryos [27]. These observations suggest that inhibition of Activin/Nodal signalling is essential for induction of neuroectoderm not only in vitro but also during gastrulation in vivo.

We also observed that addition of Noggin could improve neuroectoderm differentiation of hESCs and mEpiSCs (data not shown), suggesting that their endogenous BMP signalling interferes with neuroectoderm specification. This result reinforces studies in the mouse [28] and in amphibians [29] showing that BMP inhibits early neuronal development and thus its inhibition in turn is necessary for proper neuroectoderm specification. However in hESCs and mEpiSCs, BMP signalling acts as a potent inducer of extra-embryonic tissue differentiation (in the absence of high Activin and of FGF) and of mesendoderm progenitors (in the presence of Activin high dose and FGF). Thus, BMP signalling acts in combination with other growth factors to induce differentiation rather than to maintain pluripotency, as it does in mESCs in combination with LIF [14]. A role for BMP4 in mesendoderm specification has also been clearly demonstrated by genetic studies in the mouse showing that absence of BMP signalling arrests the development of embryos at the early gastrulation stage [30]–[32]. Consistently with this, BMP4 in combination with Activin and FGF is both necessary and sufficient to drive differentiation of hESCs and mEpiSCs into mesendoderm progenitors in vitro. BMP4 thereby appears to convert Activin/Nodal signalling into a potent inducer of mesendoderm differentiation, since when combined with Activin or with Activin+FGF2, BMP4 is able to induce differentiation of hESCs in a fully chemically defined medium. Finally, the importance of BMP4 signalling in the differentiation of extra-embryonic endoderm into visceral endoderm has been clearly demonstrated in the mouse [33], [34]. However, an essential function of BMP in the earliest stages of extraembryonic tissues specification remains intriguing since this function has not been revealed through in vivo loss of function studies [30]–[32]. Yet, those studies cannot exclude a maternal effect or signalling through other receptors, and thus further in vivo investigations are warranted to definitively exclude such a role for BMP signalling in vivo. Taken together, these observations show that Activin, BMP and FGF signalling play pivotal functions in cell fate specification of pluripotent stem cells. Moreover, the mechanisms controlled by these pathways mimic those involved in the specification of the embryonic germ layers during in vivo mammalian development. Accordingly, the growth factor-driven cell fate choices methods of differentiation described here provide an opportunity to study the molecular mechanisms controlling cell fate specification of pluripotent cells equivalent to early stages of human and mouse embryonic development.

To conclude, growing hESCs and mEpiSCs in chemically defined conditions reveals an overarching similarity in their responsiveness to growth factors that maintain pluripotency or induce differentiation. The distinction between their shared responses and those of mESCs to these same growth factors underscores the development of pluripotency itself: pluripotent phenotypes range from that of mESCs (derived from the inner cell mass of the late blastocyst) to EpiSCs (derived from pre-gastrulation stages) and hESCs. Our findings lead to the conclusion that dependency on Activin/Nodal signalling itself defines a stage of mammalian stem cell pluripotency regardless of provenance. Therefore, the further investigation of the signalling cascades responsible for pluripotency and differentiation of such pluripotent stem cells will be valuable for understanding mechanisms underlying their similarities, as well as for delivering the clinical benefits of hESCs.

## Materials and Methods

### Growth of hESCs in feeder free and serum free conditions

For feeder and serum free culture, H9 (WiCell, Madison, WI) and hSF-6 (UCSF, San Francisco, CA) hESCs were grown in chemically defined medium (CDM) [9], supplemented with Activin (10 ng/ml, RandD systems) and FGF2 (12 ng/ml, RandD systems). The original composition of CDM was 50% IMDM (Gibco) plus 50% F12 NUT-MIX (Gibco), supplemented with 7 µg/ml of insulin (Roche), 15 µg/ml of transferrin (Roche), 450 µM of monothioglycerol (Sigma) and 5 mg/ml bovine serum albumin fraction V (Europabioproducts). For the humanised version of CDM, BSA was replaced by human Serum Albumin (Sigma) or Polyvinyl Alcohol (CDM-PVA) (Sigma). Every 4 days, cells were harvested using 5 mg/ml collagenase IV (Gibco) and then plated into dishes (Costar) that were pre-coated with 15 µg/ml of human Fibronectin (Chemicon) for 20 minutes at 37°C and then washed twice in PBS.

### Differentiation of hESCs in chemically defined conditions

hESCs grown in feeder free and serum free conditions were harvested using 5 mg/ml collagenase IV or Accutase then split into plates pre-coated with fibronectin. hESCs were grown for the first two days in CDM supplemented with Activin (10 ng/ml, RandD systems) and FGF2 (12 ng/ml, RandD systems). To obtain neuroectoderm progenitors, hESCs were grown in CDM or in CDM-PVA in the presence of SB431542 (10 µM Tocris) and FGF2 (12 ng/ml, RandD systems) for 7 additional days. The resulting cells were grown in non-adherent conditions for 10 days and then plated back for 5 additional days in CDM to obtain fully differentiated neuronal cells. To obtain mesendoderm precursors, hESCs were grown for the 3 following days in CDM-PVA in the presence of SU5402 (10 µM, Calbiochem) and Activin (5 ng/ml, RandD systems). Then, the resulting cells were grown for a further 3 or 4 days in CDM-PVA in the presence of BMP4 (10 ng/ml, RandD systems), FGF2 (20 ng/ml, RandD systems) and Activin (30 ng/ml or 100 ng/ml, RandD systems).

### Differentiation of mESCs in chemically defined conditions

R1 mESCs and CGR8 mESCs were grown on 60 mm plates (Costar) pre-coated with 0.1% porcine gelatine (Sigma) and containing 1×10^5^ irradiated mouse embryonic fibroblasts in KSR medium containing 20% serum Replacer (Gibco) supplemented with LIF. Confluent cells were passaged using trypsin (Gibco) and then transferred at low density (∼1000 cells/well of a 6 well plate) on plastic plates (Costar) pre-coated with 0.1% porcine gelatine (Sigma) and FBS (Hyclone). The resulting cells were then grown in the culture conditions inductive for extra-embryonic, neuroectoderm and mesendoderm differentiation as described above.

### Flow Cytometry and cell sorting

For detection of N-CAM, CXCR4 and PDGFα Receptor, adherent cells were washed twice in PBS and then incubated for 20 minutes at 37°C in Cell Dissociation Buffer (Invitrogen). Cells were dissociated by gentle pipetting and resuspended at approximately 0.1 to 1×10^5^ cells/ml in PBS+3% normal goat serum containing 0.1% azide (NGS) (Serotec). Cells were incubated for 40 minutes at 4°C with FITC or PE conjugated antibody to CXCR4 (1∶50, Pharmingen), N-CAM (1∶200, Pharmingen), or PDGFαR (1∶50, Pharmingen) or the corresponding isotype control (mouse IgG isotype control, Pharmingen). Subsequently, cells were resuspended in PBS+3% NGS for staining with 7-aminoactinomycin D (7-AAD) viability dye (Immunotech) at 20 µl/ml for 15 minutes at room temperature. Live cells identified by 7-AAD exclusion were analyzed for surface-marker expression using FACSCalibur or sorted using a Dakocytomation Mo Flo high speed flowcytometer.

### RNA extraction and Real time PCR

Total RNAs were extracted from hESCs or differentiated progenitors using the RNeasy Mini Kit (Qiagen). Each sample was treated with RNAse-Free DNAse (Qiagen) in order to avoid DNA contamination. For each sample 0.6 µg of total RNA was reverse transcribed using Superscript II Reverse Transcriptase (Invitrogen). Real time PCR reaction mixtures were prepared as described (SensiMiX protocol Quantace) then denatured at 94°C for 5 minutes and cycled at 94°C for 30 seconds, 60°C for 30 seconds, and 72°C for 30 seconds followed by final extension at 72°C for 10 minutes after completion of 40 cycles. Primer sequences were described elsewhere[2], [25]. Real time PCR reactions were performed using a Stratagene Mx3005P in triplicate and normalised to PBGD in the same run.

### Microarray Methods

Sample RNA was hybridized to Affymetrix *hg-u133+2* GeneChips©. All sample arrays were background corrected, normalized and summarized using default parameters of the RMA model [35]. Array processing was performed using the *affy* package of the *Bioconductor* (http://www.bioconductor.org) suite of software for the *R* statistical programming language (http://www.r-project.org). The resulting data set contained processed gene expression values for 54675 probe-sets. All hybridizations employed are publicly available in MIAME format from the ArrayExpress microarray repository (European Bioinformatics Institute; http://www.ebi.ac.uk/arrayexpress) with accession number E-MEXP-930. For the Analysis of Differential Regulation, the moderated t-statistic of [36], implemented in the *Limma* package of *Bioconductor*, was employed to assess the significance of differential gene (probe-set) expression between sample groups. In order to reduce errors associated with multiple hypothesis testing on such a scale, the significance *p-values* obtained were converted to corrected *q-values* using the FDR method [37]. Probe-sets with associated *q*<0.01 (FDR 1%) were deemed to exhibit significant differential expression between sample groups. For data visualisation, the heat maps of gene expression were created by importing relevant subsets of RMA processed microarray gene expression data into the *dChip v1.3* microarray analysis package (http://www.biostat.harvard.edu/complab/dchip/). In the case wherein a gene is represented by more than one probe-set on the array, a single probe-set was chosen to represent gene expression in the heat map according to highest mean expression over all samples (i.e. the most reliable sample hybridization regardless of group membership).

### Immunofluorescence

HESCs or their differentiated progeny were fixed for 20 minutes at 4°C in 4% paraformaldehyde (PFA) and then washed three times in PBS. Cells were incubated for 20 minutes at room temperature in PBS containing 10% donkey serum (Serotec) and subsequently incubated overnight at 4°C with primary antibody diluted in 1% donkey serum in PBS as follows: Oct-4 (1∶100, Abcam ab18976 or SantaCruz), Sox2 (1∶100, Abcam ab15830), Brachyury (1∶100, Abcam ab20680 or RandD systems), Sox17 (RandD systems), FoxA2 (1∶50, Abcam ab5074), GATA4 (1∶250, SantaCruz), GATA6 (1∶200, Abcam ab22600 or Santa Cruz), Vent-2 (1∶100, Abcam ab20913), Goosecoid (1∶25, Abcam ab21059), Wnt3A (1∶100, Abcam ab19925), CXCR4 (1∶100, RandD Systems or Pharmingen). Nestin (1∶200, Abcam ab5968), NCAM (1∶100, Abcam ab8077), GFAP (Abcam), Neurofilament (Abcam), βIII Tubulin (Abcam), SMI31 (Covance), Glutamate (Sigma), N-Cadherin (1∶100, Abcam ab18203). Cells were then washed three times in PBS and incubated with Texas Red or fluorescein-Isothiocyanate-conjugated anti-mouse IgG (Sigma 1∶200 in 1% donkey serum in PBS) or rabbit IgG (Jackson laboratory 1∶400 in donkey serum in PBS) or Goat IgG (Jackson laboratory 1∶400 in donkey serum in PBS) for two hours at room temperature. Unbound secondary antibody was removed by 3 washes in PBS. Hoechst 33258 was added to the first wash (Sigma 1∶10000).
